# Supplementary material for: Non-obligate pairwise metabolite cross-feeding suggests ammensalic interactions between Bacillus amyloliquefaciens and Aspergillus oryzae
Source: Commun Biol. 2022 Mar 15;5:232. doi: 10.1038/s42003-022-03181-7 (PMC8924192; doi:10.1038/s42003-022-03181-7)
Supplement: Supplementary file 2 — Supplementary information [file 42003_2022_3181_MOESM2_ESM.pdf]

Supplementary information

Non-obligate pairwise metabolite cross-feeding suggests ammensalic interactions between *Bacillus amyloliquefaciens* and *Aspergillus oryzae*

Digar Singh[1], Sang Hee Lee[1], and Choong Hwan Lee[1]*

[1]Department of Bioscience and Biotechnology, Konkuk University, 05029, Seoul, Korea

* Corresponding author: Telephone: (+82) 220496177; Fax Number: (+82) 24454291

E-mail: chlee123@konkuk.ac.kr

Competing interest. The authors declare no competing interests.

Supplementary figures

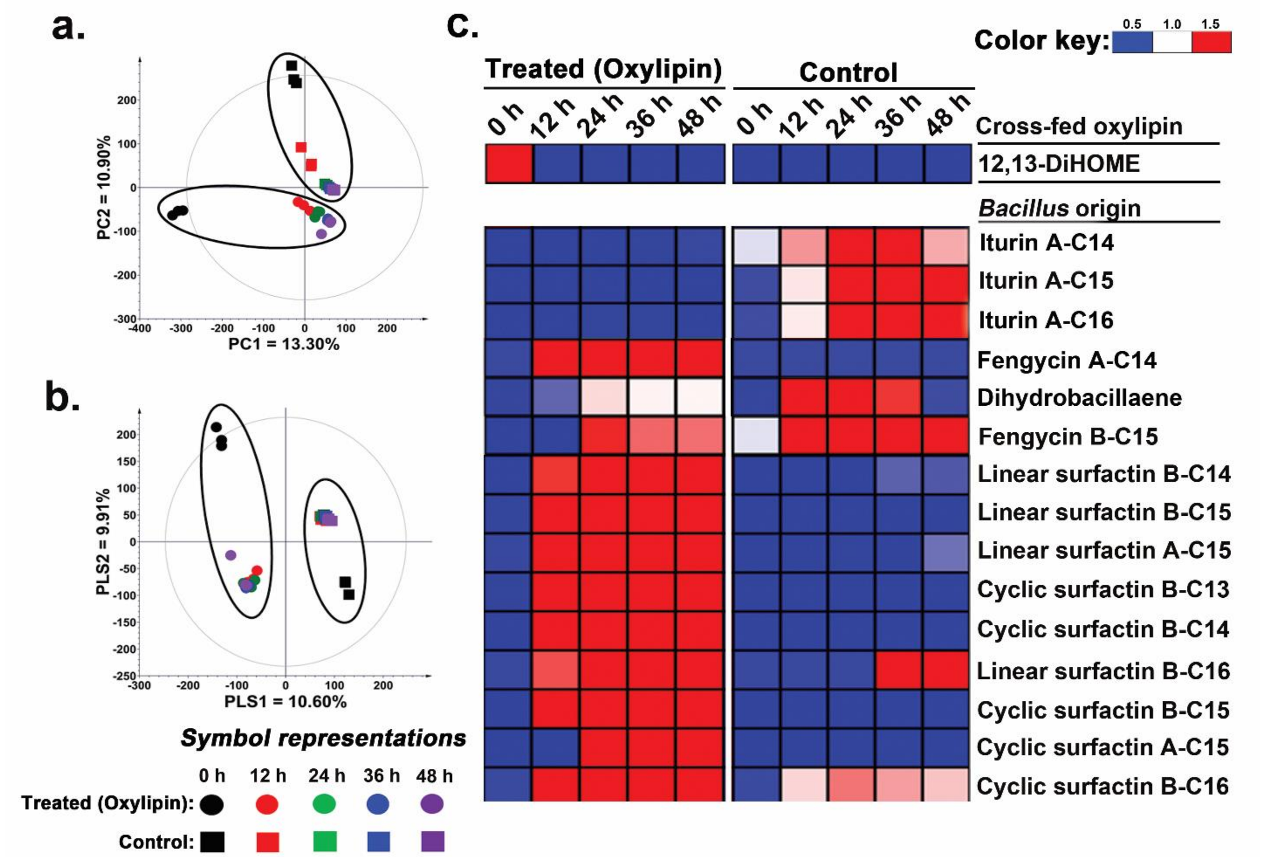

Supplementary figure 1. Time correlated **(a)** PCA, and **(b)** PLS-DA score plots representing the extracellular metabolite profiles for *B. amyloliquefaciens* cultures subjected to oxylipin 12,13-DiHOME treatment and control sets based on the non-untargeted UHPLC-LTQ-Orbitrap-MS data acquired in negative ion mode, and **(c)** the corresponding heat map based on the PLD-DA model displaying the relative abundance of the significantly discriminant metabolites between oxylipin treated and control sets. The corresponding source data files for figures 1.a & b and 1.c can be found at sheet 2 and 3, respectively, in supplementary data 4.

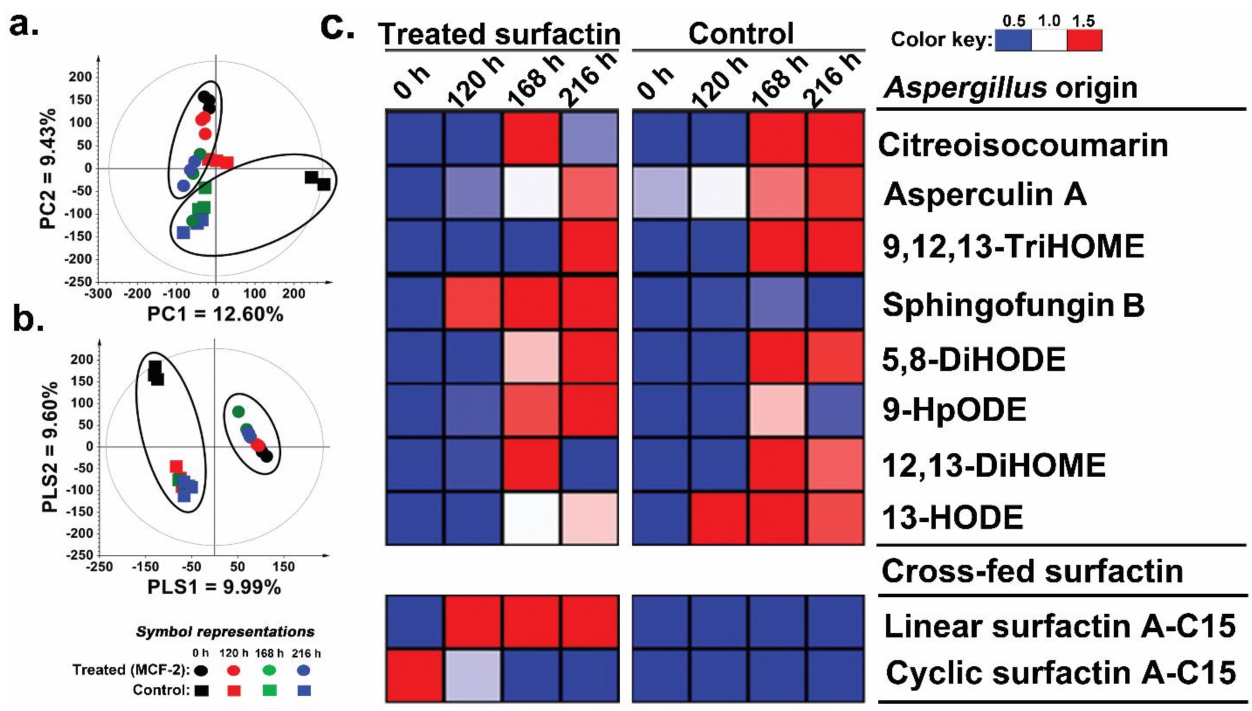

Supplementary figure 2. Time correlated **(a)** PCA, and **(b)** PLS-DA score plots depicting marked variations between the metabolite profiles of cyclic surfactin A-C15 treated and control *A. oryzae* culture extracts based on the non-untargeted UHPLC-LTQ-Orbitrap-MS datasets acquired in negative ion mode, and **(c)** the corresponding heat map based on the PLD-DA model displaying the relative abundance of the significantly discriminant metabolites between the surfactin treated and control culture extracts. The corresponding source data files for figures 2.a & b and 2.c can be found at sheet 2 and 3, respectively, in supplementary data 5.

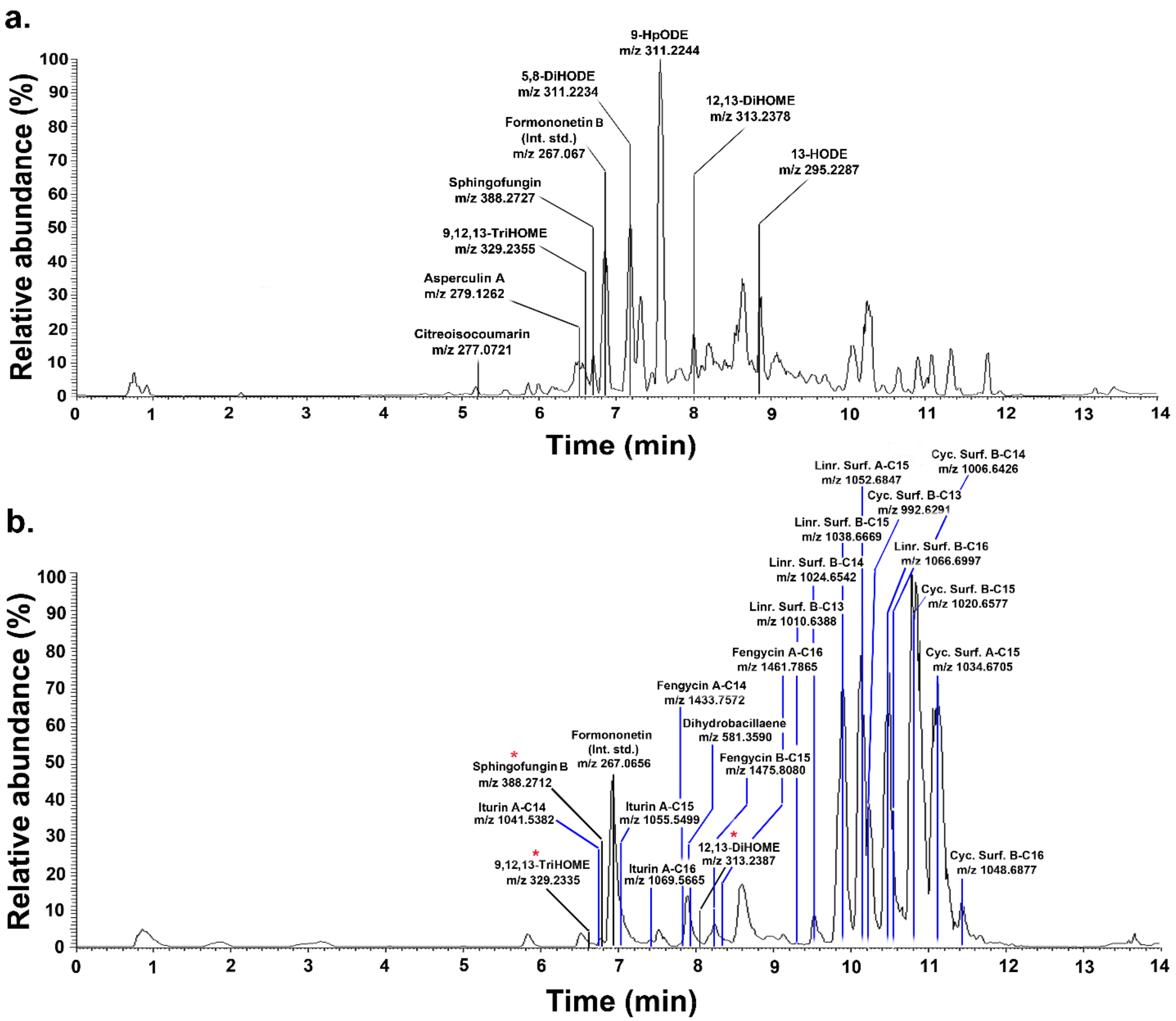

Supplementary figure 3. The UHPLC-LTQ-Orbitrap-MS/MS chromatograms representing the QC (quality control) mixtures for **(a)** the late-log phase *A. oryzae* (A_d; donor species) culture extracts used in the metabolite cross feeding, and (b) the *B. amyloliquefaciens* (B_r; receiver species) culture extracts after MCF-1 ($A_d \rightarrow B_r$). Here, the cross-fed metabolites of *Aspergillus* origin which were re-extracted from *Bacillus* cultures are highlighted with red asterisks (*) in the chromatogram b. Only the annotated metabolites are shown while the non-identified metabolites are not indicated in the chromatogram.

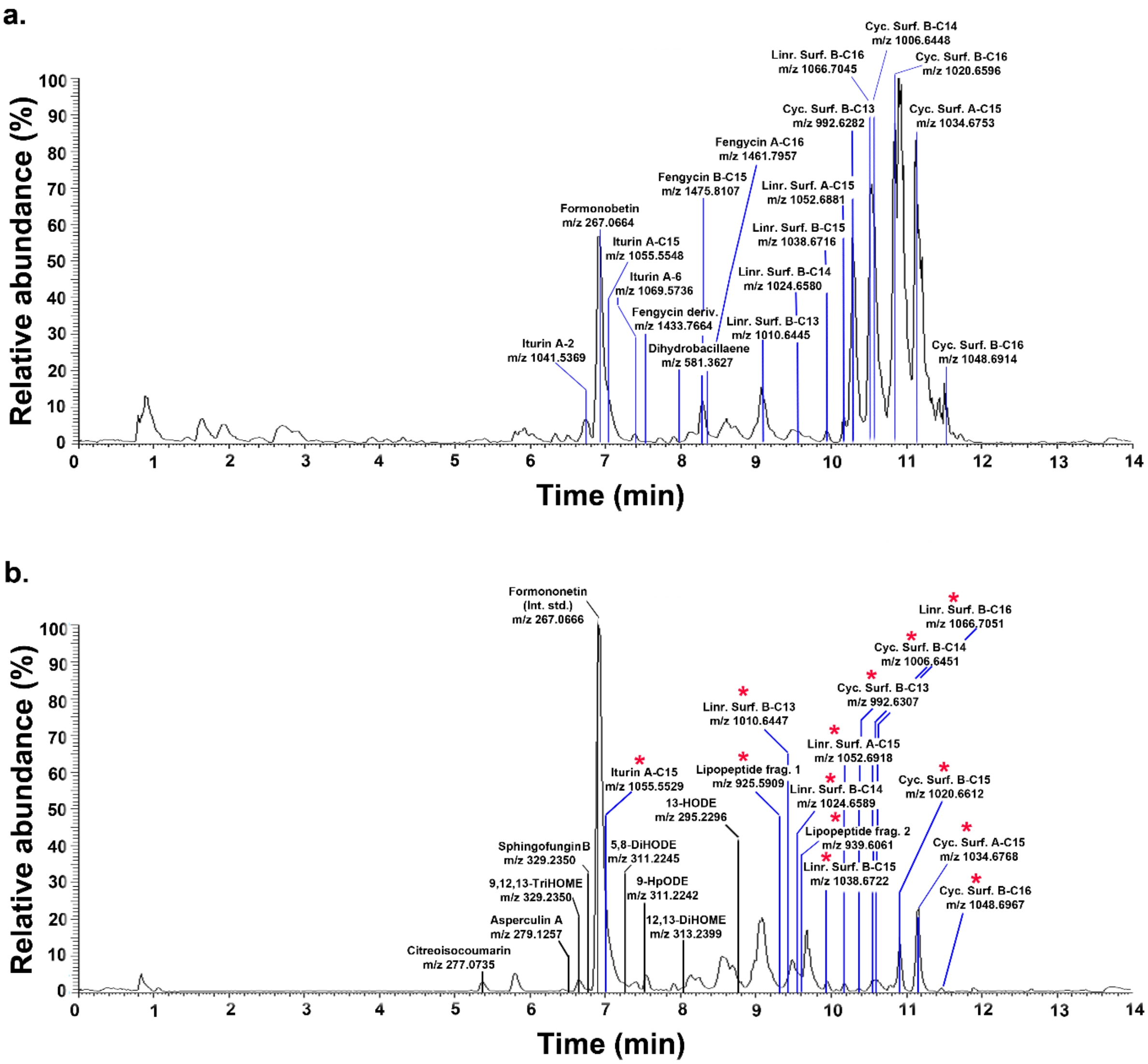

Supplementary figure 4. The UHPLC-LTQ-Orbitrap-MS/MS chromatograms representing the QC (quality control) mixtures for **(a)** the late-log phase *B. amyloliquefaciens* (B_d; donor species) culture extracts used in the metabolite cross feeding, and (b) the *A. oryzae* (A_r; receiver species) culture extracts after MCF-2 ($B_d \rightarrow A_r$). Here, the cross-fed metabolites of *Bacillus* origin which were re-extracted from *Aspergillus* cultures are highlighted with red asterisks (*) in the chromatogram b. Only the annotated metabolites are shown while the non-identified metabolites are not indicated in the chromatogram.

Supplementary tables

Supplementary table 1. List of the significantly discriminant metabolites (VIP>0.7 and *p*<0.05) and their chromatographic & spectral characteristics based on the LC-MS analysis obtained for MCF-1 treated ($A_d \rightarrow B_r$) and control sets of *B. amyloliquefaciens*.

S. No.	Metabolites	RT	m/z (-ESI)	m/z (+ESI)	Mol. Wt.	Mass Fragmentation	Elemental composition	Δm (ppm)	References
**Cross-fed and re-extracted metabolites of *A. oryzae* origin**									
1	9,12,13-TriHOME	6.62	329.23	ND	330	(-) 329>311, **293**, 229, 211, 171	$C_{18}H_{34}O_5$	0.07	[1]
2	Sphingofungin B	6.79	388.27	ND	389	(-) 388>**370**, 346>352, 328>**310**, 293, 285, 267	$C_{20}H_{39}O_6N$	0.74	[2]
3	12, 13-DiHOME	8.06	313.24	ND	314	(-) 313>**295**, 283, 267, 183	$C_{18}H_{34}O_4$	1.27	[1], Std. comp.
**Endogenous metabolites of *B. amyloliquefaciens* origin**									
4	Iturin A-C14	6.77	1041.54	1043.55	1042	(-) 1041>1023, **1011**>994, **993**>977, 922, **822**	$C_{48}H_{74}O_{14}N_{12}$	-0.34	[3, 4]
5	Iturin A-C15	7.01	1055.55	1057.57	1056	(-) 1056>1037, **1025**, 1019>**1008**>1009, **990**, 965	$C_{49}H_{76}O_{14}N_{12}$	-0.48	[4]
6	Iturin A-C16	7.36	1069.57	1071.58	1070	(-) 1069>**1052**, 1040>**1022**>**1005**	$C_{50}H_{78}O_{14}N_{12}$	-0.95	[5]
7	Fengycin A-C14	7.79	1433.75	1435.78	1435	(-) 1434>**1390**>1373, **1346**>**1328**, 1199, 1144, 1102	$C_{70}H_{106}O_{20}N_{12}$	-0.80	[6]
8	Dihydrobacillaene	7.96	581.36	ND	582	(-) 537, 519, **358**>**340**, 330, 300> 322, 312, 254, **226**	$C_{34}H_{50}O_6N_2$	-1.44	PubChem
9	Fengycin B-C15	8.24	1475.80	1477.82	1477	(-) 1476>1458, **1432**>1414, **1388**>1370, 1326, **1176**,	$C_{73}H_{112}O_{20}N_{12}$	-1.31	[6]
10	Fengycin C-C16	8.37	1461.78	1463.81	1463	(-) 1462>1433, **1417**>1400, **1374**>1356, **1144**, 1102	$C_{72}H_{110}O_{20}N_{12}$	0.28	[6]
11	Linear surfactin B-C13	9.29	1010.64	1012.66	1012	(-) 1011>**993**>**975**>957, **818**, 775	$C_{50}H_{89}O_{14}N_7$	-0.68	Theoretical
12	Linear surfactin B-C14	9.52	1024.65	1026.67	1026	(-) 1025>1008, **989**, 971, 819>776, **664**, 452	$C_{51}H_{91}O_{14}N_7$	-0.93	Theoretical
13	Linear surfactin B-C15	9.90	1038.67	1040.69	1040	(-) 1039>**1021**>**1003**>984, **818**, 776, 696, 678, 452	$C_{52}H_{93}O_{14}N_7$	-3.79	Theoretical
14	Linear surfactin A-C15	10.11	1052.68	1054.70	1054	(-) 1053>**1035**>**1017**>999, 888, **818**	$C_{53}H_{95}O_{14}N_7$	-5.70	Theoretical
15	Cyclic surfactin B-C13	10.22	992.63	994.64	994	(+) 995>977, 881, **685**>667, 554, **441**>413, **328**	$C_{50}H_{87}O_{13}N_7$	-0.83	[7]
16	Linear surfactin B-C16	10.43	1066.70	1068.72	1068	(-) 1067>**1049**>**1031**>1013, **819**, 776	$C_{54}H_{97}O_{14}N_7$	-4.85	Theoretical
17	Cyclic surfactin B-C14	10.46	1006.64	1008.66	1008	(-) 1007>**989**>971, 927, 776, **665**>**452**, 339/ (+) 1009>991, **685**>667, 554, **441**>227, **199**	$C_{51}H_{89}O_{13}N_7$	-2.50	[8]
18	Cyclic surfactin B-C15	10.82	1020.66	1022.67	1022	(-) 1021>**1003**>**985**, 941, 776, 678>**422**	$C_{52}H_{91}O_{13}N_7$	-2.45	[7]
19	Cyclic surfactin A-C15	11.12	1034.67	1036.69	1036	(-) 1035>**1017**>**999**>355, 790, **692**, 466	$C_{53}H_{93}O_{13}N_7$	-5.86	Std. comp.
20	Cyclic surfactin B-C16	11.41	1048.69	1050.70	1050	(+) 1051>1033, 938, **685**>667, 554, **441**>413, **328**	$C_{54}H_{95}O_{13}N_7$	-1.66	[9]
**Non-identified metabolites (N.I)**									
21	N.I. 1 (*Aspergillus* origin)	6.22	420.15	ND	421	(-) 420>**269**>223, **187**, 144, 124	NA	NA	NA
22	N. I. 2	3.07	295.18	297.19	296	(-) 295>251, **207**>**179**, 151>**164**, 136	NA	NA	NA
23	N. I. 3	5.70	377.18	ND	278	(-) 377>359, **320**>303, 277, **206**>**192**>**178**	NA	NA	NA
24	N. I. 4	6.34	316.21	ND	317	(-) 316>**286**, 272>**242**>**226**, 170	NA	NA	NA
25	N. I. 5	6.51	609.29	ND	610	(-) 609>**563**, 541>519, **495**>427, **408**, 385	NA	NA	NA
26	N.I. 6 (*Bacillus* origin)	7.39	588.28	* 612.27	589	(-) 588>552, 433, 305, **186**>167, **142**>**124**	NA	NA	NA
27	N.I. 7 (*Bacillus* origin)	7.23	741.40	743.41	742	(-) 741>679, 559, 518>500, 460, 434, **356**>**338**, 242	NA	NA	NA
28	N. I. 8	8.13	438.26	ND	439	(-) 438>297, **241**>**223**>**81**	NA	NA	NA

- **RT:** Retention time; **Δm:** mass error; * [M + Na^+ adduct]; **ND:** not detected; **NA:** not applicable; **Std. comp:** verified with standard compound.
- Bold numbers in the mass fragmentation column indicates the major peaks.

- (-)/ (+) in the mass fragmentation column indicates the ESI modes in which the data were recorded for respective peaks.

Supplementary table 2. List of the significantly discriminant metabolites (VIP>0.7 and *p*<0.05) and their chromatographic & spectral characteristics based on the LC-MS analysis obtained for MCF-2 treated ($B_d \rightarrow A_r$) and control sets of *A. oryzae*.

S. No.	Candidate metabolites	RT	m/z (-ESI)	m/z (+ESI)	Mol. Wt.	Mass Fragmentation	Elemental composition	Δm (ppm)	References
**Endogenous metabolites of *A. oryzae* origin**									
1	Citreoisocoumarin	5.30	277.07	279.09	278.00	(-)277>233, **219**>191>147, **123**	$C_{14}H_{14}O_6$	6.24	[10]
2	Asperaculin A	6.51	279.13	ND	280.00	(-)279>235, **205**>187, 177, **149**>**106**	$C_{18}H_{20}O_5$	3.02	[10]
3	9,12,13-TriHOME	6.60	329.24	ND	330.00	(-)311> **293**, 229, 211>153, 127, **171**	$C_{18}H_{34}O_5$	8.50	[1]
4	Sphingofungin B	6.76	388.27	390.28	389.00	Fragmentation not observed	$C_{20}H_{39}O_6N$	7.67	[2]
5	5,8-DiHODE	7.22	311.22	ND	312.00	(-)311>**293**>275, **249**>257, **231**, 177	$C_{18}H_{32}O_4$	1.75	[1], Std. comp.
6	9-HpODE	7.35	311.22	ND	312.00	(-)311>**293**, 267>275, **249**>231, 205, 156, **113**	$C_{18}H_{32}O_4$	1.31	[11]
7	12,13-DiHOME	8.05	313.24	ND	314.00	(-)313>298, **295**>281, **277**>**259**, 249	$C_{18}H_{34}O_4$	1.41	[1], Std. comp.
8	13-HODE	8.94	295.23	ND	296.00	(-)295>**277**, 251>259, **233**>**191**, 179	$C_{18}H_{32}O_3$	9.51	[11]
**Cross-fed and re-extracted metabolite of *B. amyloliquefaciens* origin**									
9	Iturin A-C15	7.00	1055.56	ND	1057.00	Fragmentation not observed	$C_{49}H_{76}O_{14}N_{12}$	2.65	[4]
10	Lipopeptide frag. 1	9.31	925.59	927.60	926.00	(-)926>**907**, 795, 697> **890**, 697, 663, 452, 339>434, **240**	ND	NA	NA
11	Linear surfactin B-C13	9.32	1010.65	1012.65	1012.00	(-)1011>**993**>**975**>956, **819**, 776, 669, 452	$C_{50}H_{89}O_{14}N_7$	3.74	Theoretical
12	Linear surfactin B-C14	9.56	1024.66	1026.67	1026.00	(-)1025>**1008**, 989>836, **819**, 683>777, 683	$C_{51}H_{91}O_{14}N_7$	2.17	Theoretical
13	Lipopeptide frag. 2	9.56	939.61	941.62	941.00	(-)940>922, **809**, 711>**692**, 512, 452, 339>**434**, 240, 222	ND	NA	NA
14	Linear surfactin B-C15	9.94	1038.68	1040.69	1040	(-)1039>**1021**>**1003**>985, **819**, 777, 696, 452	$C_{52}H_{93}O_{14}N_7$	1.30	Theoretical
15	Linear surfactin A-C15	10.17	1052.69	1054.70	1054.00	(-)1053>**1035**>**1017**>**999**, 887, **818**, 776, 711, 693, 452	$C_{53}H_{95}O_{14}N_7$	0.91	Theoretical
16	Cyclic Surfactin B-C13	10.29	992.63	ND	994.00	(-)993>**975**>956, **913**, 794>776, **650**, 452	$C_{50}H_{87}O_{13}N_7$	2.27	[7]
17	Cyclic Surfactin B-C14	10.53	1006.65	1008.66	1008.00	(-)1007>**989**>971, 777, **665**>**452**, 339	$C_{51}H_{89}O_{13}N_7$	1.49	[8]
18	Linear surfactin B-C16	10.55	1066.71	ND	1068.00	Fragmentation not observed	$C_{54}H_{97}O_{14}N_7$	3.96	Theoretical
19	Cyclic Surfactin B-C15	10.88	1020.66	1022.67	1022.00	(-)1021>**1003**>985, 777, 679, 452	$C_{52}H_{91}O_{13}N_7$	2.58	[7]
20	Cyclic Surfactin A-C15	11.18	1034.68	1036.69	1036.00	(-)1035>**1017**>999, 777, **693**>452, **339**	$C_{53}H_{93}O_{13}N_7$	2.40	Std. comp.
21	Cyclic surfactin B-C16	11.29	1048.69	1050.70	1050.00	Fragmentation not observed	$C_{54}H_{95}O_{13}N_7$	5.14	[9]
**Non-identified metabolites (N.I)**									
22	N.I. 1	8.30	491.27	493.28	492.00	(-)473, **447**, 400, 344, 326, 262, 227>283, 194, 179>164	NA	NA	NA
23	N.I. 2	8.54	487.34	ND	488.00	(-)487>**469**, 427, 397>425, 407, 383>392, 337	NA	NA	NA

RT: Retention time; **Δm:** mass error

- Bold numbers in the mass fragmentation column indicates the major peaks.
- **ND:** not detected
- **NA:** not applicable

Supplementary references

1. Singh, D, & Lee, C. H. Volatiles mediated interactions between *Aspergillus oryzae* strains modulate morphological transition and exometabolomes. *Front. Microbiol.* **9**, 628 (2018).
2. Sud, M., Fahy, E., Cotter, D., Dennis, E. A. & Subramaniam, S. LIPID MAPS-Nature Lipidomics Gateway: An online Resource for Students and Educators Interested in Lipids. *J. Chem. Educ.* **89**, 291-92 (2012).
3. Pathak, K. V. & Keharia, H. Identification of surfactins and iturins produced by potent fungal antagonist, *Bacillus subtilis* K1 isolated from aerial roots of banyan (*Ficus benghalensis*) tree using mass spectrometry. *3 Biotech* **4**, 283-95 (2014).
4. Zhang, Q. X., Zhang, Y., Shan, H. H., Tong, Y. H, Chen, X. J. & Liu, F. Q. Isolation and identification of antifungal peptides from *Bacillus amyloliquefaciens* W10. Environ. *Sci. Pollut. Res. Int.* **24**, 25000-9 (2017).
5. Dang, Y. et al. Enhanced production of antifungal lipopeptide iturin A by *Bacillus amyloliquefaciens* LL3 through metabolic engineering and culture conditions optimization. *Microb. Cell. Fact.* **18,** 1-14 (2019).
6. Li, X. Y., Mao, Z. C., Wang, Y. H., Wu, Y. X., He, Y. Q. & Long, C. L. ESI LC-MS and MS/MS Characterization of antifungal cyclic lipopeptides produced by *Bacillus subtilis* XF-1. *J. Mol. Microbiol. Biotechnol.* **22**, 83-93 (2012).
7. Deng, Q. et al. A sensitive method for simultaneous quantitative determination of surfactin and iturin by LC-MS/MS. *Anal. Bioanal. Chem.* **409**, 179-91 (2017).
8. Perez, K. J. et al. *Bacillus* spp. isolated from Puba as a source of biosurfactants and antimicrobial lipopeptides. *Front. Microbiol.* **8**, 61 (2017).
9. Janek, T. et al. Sustainable Surfactin Production by *Bacillus subtilis* Using Crude Glycerol from Different Wastes. *Molecules* **26,** 3488 (2021).

10. Son, S. Y., Lee, S., Singh, D., Lee, N. R., Lee, D. Y. & Lee C. H. Comprehensive secondary metabolite profiling toward delineating the solid and submerged-state fermentation of *Aspergillus oryzae* KCCM 12698. *Front. Microbiol.* **9**, 1076 (2018).

11. Guijas, C. et al. METLIN: a technology platform for identifying knowns and unknowns. *Anal. Chem.* **90**, 3156-3164 (2018).
