# Supplementary material for: Non-obligate pairwise metabolite cross-feeding suggests ammensalic interactions between Bacillus amyloliquefaciens and Aspergillus oryzae
Source: Commun Biol. 2022 Mar 15;5:232. doi: 10.1038/s42003-022-03181-7 (PMC8924192; doi:10.1038/s42003-022-03181-7)
Supplement: Supplementary file 3 — Description of Additional Supplementary Files [file 42003_2022_3181_MOESM3_ESM.pdf]

Description of Additional Supplementary Files

File name: Supplementary Data 1-6
Description: Source data for microbial phenotypes, LC-MS/MS metabolite profiling, and associated statistical correlations.
