# Supplementary material for: Non-obligate pairwise metabolite cross-feeding suggests ammensalic interactions between Bacillus amyloliquefaciens and Aspergillus oryzae
Source: Commun Biol. 2022 Mar 15;5:232. doi: 10.1038/s42003-022-03181-7 (PMC8924192; doi:10.1038/s42003-022-03181-7)
Supplement: Supplementary file 10 — Reporting Summary [file 42003_2022_3181_MOESM10_ESM.pdf]

nature research

Corresponding author(s): Choong Hwan Lee (Manuscript ID: COMMSBIO-21-2493A)

Last updated by author(s): Jan 28, 2022

Reporting Summary

Nature Research wishes to improve the reproducibility of the work that we publish. This form provides structure for consistency and transparency in reporting. For further information on Nature Research policies, see our Editorial Policies and the Editorial Policy Checklist.

Statistics

For all statistical analyses, confirm that the following items are present in the figure legend, table legend, main text, or Methods section.

n/a	Confirmed	
☐	☒	The exact sample size (*n*) for each experimental group/condition, given as a discrete number and unit of measurement
☐	☒	A statement on whether measurements were taken from distinct samples or whether the same sample was measured repeatedly
☐	☒	The statistical test(s) used AND whether they are one- or two-sided<br>*Only common tests should be described solely by name; describe more complex techniques in the Methods section.*
☐	☒	A description of all covariates tested
☐	☒	A description of any assumptions or corrections, such as tests of normality and adjustment for multiple comparisons
☐	☒	A full description of the statistical parameters including central tendency (e.g. means) or other basic estimates (e.g. regression coefficient) AND variation (e.g. standard deviation) or associated estimates of uncertainty (e.g. confidence intervals)
☒	☐	For null hypothesis testing, the test statistic (e.g. *F*, *t*, *r*) with confidence intervals, effect sizes, degrees of freedom and *P* value noted<br>*Give P values as exact values whenever suitable.*
☒	☐	For Bayesian analysis, information on the choice of priors and Markov chain Monte Carlo settings
☒	☐	For hierarchical and complex designs, identification of the appropriate level for tests and full reporting of outcomes
☐	☒	Estimates of effect sizes (e.g. Cohen's *d*, Pearson's *r*), indicating how they were calculated

Our web collection on statistics for biologists contains articles on many of the points above.

Software and code

Policy information about availability of computer code

Data collection	No software was used to collect the data for this study.
Data analysis	1. Thermor Xcalibur 2.2, Waltham, MA, USA was used for LC-MS data analysis and raw file (CDF) conversion.<br>2. MetAlign v.041012, RIKILT-WUR, Institute of Food Safety was used for LC-MS data alignment for significant peak-picking, mass artifact filtration, baseline correction, RT shift corrections, and accurate mass calculation.<br>3. SIMCA-P+ v 12.0, Umetrics, Umea, Sweden was used toward multivariate analyses (PCA and PLS-DA) of the metabolomics data.<br>4. PASW statistics (SPSS Inc. Chicago, Illinois, USA) was used for calculating bivariate pearson's correlations between the metabolomics and phenotype data.<br>5. Cytoscape software v 3.7.2 was used for visualizing the correlation networks<br>6. Sigma plot (SPSS) v 10.2 was used for the analyses and plotting of phenotype data.

For manuscripts utilizing custom algorithms or software that are central to the research but not yet described in published literature, software must be made available to editors and reviewers. We strongly encourage code deposition in a community repository (e.g. GitHub). See the Nature Research guidelines for submitting code & software for further information.

Data

Policy information about availability of data

All manuscripts must include a data availability statement. This statement should provide the following information, where applicable:

- Accession codes, unique identifiers, or web links for publicly available datasets
- A list of figures that have associated raw data
- A description of any restrictions on data availability

LC-MS/MS data related to this study is available from the corresponding author upon reasonable request. Source data for microbial phenotypes, LC-MS/MS metabolite profiling, and associated statistical correlations are presented in raw dataset (Excel format) files 1-4.

Field-specific reporting

Please select the one below that is the best fit for your research. If you are not sure, read the appropriate sections before making your selection.

☐ Life sciences ☐ Behavioural & social sciences ☒ Ecological, evolutionary & environmental sciences

For a reference copy of the document with all sections, see nature.com/documents/nr-reporting-summary-flat.pdf

Ecological, evolutionary & environmental sciences study design

All studies must disclose on these points even when the disclosure is negative.

Study description	It is believed that the auxotrophies determine microbial interactions through metabolite exchange, however the BFIs become hard to predict, measure, and interpret quantitatively under the nutrient rich (non-obligate) conditions involving prototrophs. To probe such BFIs, we designed a non-obligate pairwise metabolite cross-feeding (MCF) between Bacillus and Aspergillus species to deconvolute their complex metabolite trade-offs and its non-nutritional impacts on phenotypes (Bacteria: cell growth & biofilm; Fungi: mycelial growth & conidiation). Using the non-targeted metabolomics, multivariate statistics, and phenotype guided correlations, we zeroed upon the key metabolite classes from Aspergillus (oxylipins) and Bacillus (cyclic lipopeptides or CLPs) partners which modulates their ammensalic interactions. The oxylipins were observed to have signaling functions which promote growth and biofilm formation in Bacillus, while the CLPs displayed antibiosis against Aspergillus. All experiments were performed maintaining three independent biological replicates (n =3) for metabolomic analysis and microbial growth phenotypes. The microbial fitness functions or growth phenotypes were measured using complementary approaches, i.e., for bacterial growth, we examined cell's turbidity (O.D. 600nm), viability (C.F.U/mL), and biomass (C.D.W/ mL) along with biofilm formation (where 3 biological & 3 analytical replicates were recorded). Similarly, complementary growth phenotypes (conidia formation & mycelial growth) were evaluated for fungal fitness following the cross-feeding treatment. We believe that the metabolomics-based experimental & reductionist pipeline used in this study can be leveraged to design, manipulate, and understand BFIs in more complex microbiomes vital in food & nutrition, health, agriculture, and environment.
Research sample	Wild type bacterial and fungal species were used in the study. Bacillus amyloliquefaciens KCCM 43033 was procured from the 'Korean Culture Center of Microorganisms' (KCCM), Seoul, Republic of Korea. Aspergillus oryzae RIB 40 (KACC 44967), was provided by the 'Korean Agricultural Culture Collection' (KACC). Importance of recurring Bacillus and Aspergillus interactions cannot be understated in the soy food fermentations and hence understanding the complexity of metabolite mediated cross-feeding interactions between Bacillus and Aspergillus species would be critical toward the design and tractability of a scalable bioprocess. The choice of wild type (prototrophs) bacterial and fungal partners was essential toward examining the non-nutritional interactions of microbial species beyond the auxotrophies. This choice of microbial species was essential to test our hypothesis which conjectures that the lack of nutritional dependencies, more likely among the prototrophs colonizing a nutrient-rich environment, could result in a non-obligate and transient interaction.
Sampling strategy	All experiments were performed maintaining three independent biological replicates (n =3) for metabolomic analysis and microbial growth phenotypes. The microbial fitness functions or growth phenotypes were measured using complementary approaches which involved examining multiple parameters (for bacteria - O.D. 600, C.F.U/mL & C.D.W/mL; for fungi - conidia/mL & mycelial dry weight/ mL). We considered making three biologicals replicates (treated and control sets) as the biological variability is substantially greater than technical or analytical variability (Blainey et al., 2014. Nature Methods, volume 11, 879–880). We chose to make three independent biological replicates based on the literatures describing the similar in vitro studies involving microbial interactions (Clark et al., 2021, Nat Comm. 12:3254; Zhang et al., 2018, Nat Comm. 9:336).
Data collection	We performed an in vitro study where the treated (cross-fed) and control experimental sets were temporally harvested at different regular time intervals. For bacterial cultures, growth was evaluated at every 12 h intervals for variations in culture turbidity, viability, and biomass. In addition, we examined the biofilm formation as a measure of the coordinated developmental response of Bacillus towards cross-feeding treatment. The fungal cultures with longer growth duration were harvested at 0, 120, 168, and 216 h to evaluate mycelial growth and conidiation patterns. Both the treated and control samples were incubated under the same conditions and the data represent the mean (±SD) of the values corresponding to three independent biological replicates used in the study. The harvested samples were also used for metabolomic studies alongside phenotype analyses. The first and second authors of this study maintained, harvested, and recorded the experimental data using the methods described in details in this manuscript.
Timing and spatial scale	The present study is based on the in vitro experiment performed under laboratory conditions. The frequency of sample harvest were varying for the bacterial and fungal cultures owing to their different growth rates and metabolism. The bacterial samples were harvested at every 12 h intervals owing to their short-spanned (48 h) growth curve. However, the fungal samples were harvested at relatively longer intervals (0, 120, 168, and 216 h) for their slower growth rates. The choice of sample harvesting for metabolite

	extraction toward cross-feeding was made according to their respective late-log stages (Bacillus - 24-36 h; Fungi - 168-216 h). The experiment did not involve any spatial variables.
Data exclusions	We excluded the data related to initial growth curves and stability of microbial cultures in formulated medium. This was excluded as the results shown in this study were not directly related to the factors (treatment) and variables (fitness functions) described in the design of study. This exclusion was pre-established as these were the preliminary experiments to select the study parameters and not the outcomes necessary to test the hypothesis stated in the study.
Reproducibility	We expressed the experimental findings based on the data from three independent biological replicates which indicates the data reproducibility against intrinsic variations. Independent to this experiment, we have verified the robustness and reproducibility of the results shown in this study in different cultivation systems, i.e., rice koji, where the similar interactions were validated between Bacillus and Aspergillus species. However, the study on rice koji is still undergoing and we cannot publish those results alongside this study.
Randomization	Randomization for in vitro microbial experiment was not applicable in the present study. However, we randomized the samples for metabolomic analysis where the LC-MS was performed in blocks of 10 runs followed by an intermittent QC (quality control sample, with 10 µL pooled blends from all samples) run. The microbial sample extracts were randomized in each block.
Blinding	Experimental blinding for in vitro microbial experiment was not applicable in the present study.

Did the study involve field work? ☐ Yes ☒ No

Reporting for specific materials, systems and methods

We require information from authors about some types of materials, experimental systems and methods used in many studies. Here, indicate whether each material, system or method listed is relevant to your study. If you are not sure if a list item applies to your research, read the appropriate section before selecting a response.

Materials & experimental systems

n/a	Involved in the study
☒	☐ Antibodies
☒	☐ Eukaryotic cell lines
☒	☐ Palaeontology and archaeology
☒	☐ Animals and other organisms
☒	☐ Human research participants
☒	☐ Clinical data
☒	☐ Dual use research of concern

Methods

n/a	Involved in the study
☒	☐ ChIP-seq
☒	☐ Flow cytometry
☒	☐ MRI-based neuroimaging
